# Dopamine depletion and subcortical dysfunction disrupt cortical synchronization and metastability affecting cognitive function in Parkinson's disease

**DOI:** 10.1002/hbm.25745

**Published:** 2021-12-14

**Authors:** Linbo Wang, Cheng Zhou, Wei Cheng, Edmund T. Rolls, Peiyu Huang, Ningning Ma, Yuchen Liu, Yajuan Zhang, Xiaojun Guan, Tao Guo, Jingjing Wu, Ting Gao, Min Xuan, Quanquan Gu, Xiaojun Xu, Baorong Zhang, Weikang Gong, Jingnan Du, Wei Zhang, Jianfeng Feng, Minming Zhang

**Affiliations:** ^1^ Institute of Science and Technology for Brain‐Inspired Intelligence Fudan University Shanghai China; ^2^ Key Laboratory of Computational Neuroscience and Brain‐Inspired Intelligence (Fudan University) Ministry of Education China; ^3^ MOE Frontiers Center for Brain Science Fudan University Shanghai China; ^4^ Zhangjiang Fudan International Innovation Center Shanghai China; ^5^ Department of Radiology, The Second Affiliated Hospital Zhejiang University School of Medicine Hangzhou China; ^6^ Department of Computer Science University of Warwick Coventry UK; ^7^ Department of Neurology, The Second Affiliated Hospital Zhejiang University School of Medicine Hangzhou China

**Keywords:** cognition, levodopa, magnetic resonance imaging, metastability, Parkinson's disease, synchronization

## Abstract

Parkinson's disease (PD) is primarily characterized by the loss of dopaminergic cells and atrophy in subcortical regions. However, the impact of these pathological changes on large‐scale dynamic integration and segregation of the cortex are not well understood. In this study, we investigated the effect of subcortical dysfunction on cortical dynamics and cognition in PD. Spatiotemporal dynamics of the phase interactions of resting‐state blood‐oxygen‐level‐dependent signals in 159 PD patients and 152 normal control (NC) individuals were estimated. The relationships between subcortical atrophy, subcortical–cortical fiber connectivity impairment, cortical synchronization/metastability, and cognitive performance were then assessed. We found that cortical synchronization and metastability in PD patients were significantly decreased. To examine whether this is an effect of dopamine depletion, we investigated 45 PD patients both ON and OFF dopamine replacement therapy, and found that cortical synchronization and metastability are significantly increased in the ON state. The extent of cortical synchronization and metastability in the OFF state reflected cognitive performance and mediates the difference in cognitive performance between the PD and NC groups. Furthermore, both the thalamic volume and thalamocortical fiber connectivity had positive relationships with cortical synchronization and metastability in the dopaminergic OFF state, and mediate the difference in cortical synchronization between the PD and NC groups. In addition, thalamic volume also reflected cognitive performance, and cortical synchronization/metastability mediated the relationship between thalamic volume and cognitive performance in PD patients. Together, these results highlight that subcortical dysfunction and reduced dopamine levels are responsible for decreased cortical synchronization and metastability, further affecting cognitive performance in PD. This might lead to biomarkers being identified that can predict if a patient is at risk of developing dementia.

## Graphical abstract

A phase‐based cortical synchronization/metastability analysis was proposed, and decreased cortical synchronization and metastability was found in Parkinson's disease (PD). Levodopa normalized the decreased cortical synchronization and metastability in PD. Cortical synchronization and metastability decline mediated cognitive decline of PD. Thalamus volume and thalamocortical fiber connectivity mediated synchronization decline.

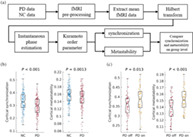

## 1 INTRODUCTION

Parkinson's disease (PD) is characterized by aggregation of degenerated α‐synuclein in the substantia nigra pars compacta and striatal dopaminergic deficiency, resulting in the cardinal motor symptoms associated with PD (Aarsland et al., 2017). However, subcortical dysfunction cannot directly account for the spectrum of non‐motor symptoms observed in PD, such as cognitive impairment, which is one of the more debilitating of symptoms with important ramifications for quality of life (Aarsland et al., 2017; Szeto, Walton, Rizos, & Martinez‐Martin, 2020). Cognitive impairment in PD does not merely involve neurotransmitter deficits, which is also characterized by altered networks of brain structures as aggregation of degenerated α‐synuclein spreads from subcortical to cortical regions (Pandya et al., 2019; Yau, Zeighami, Baker, Larcher, & Vainik, 2018). Studies in PD patients with mild cognitive impairment demonstrate dysfunction in frontal and parietal regions (Pereira et al., 2015; Suo et al., 2021). However, cognitive impairment arises before cortical thinning occurs in PD (Oxtoby et al., 2021).

One possible explanation for cognitive impairment in early PD is that pathological changes in the subcortex affect subcortical–cortical macro‐circuits that connect the basal ganglia, thalamus and cortex (i.e., the cortico–basal ganglia–thalamic [CBG] “loop”), which are involved in large‐scale network communication (Shine, Hearne, et al., 2019; Tinkhauser et al., 2018). A dynamic balance between functional integration and segregation of large‐scale brain networks is essential for a range of cognitive processes (Alderson, Bokde, Kelso, Maguire, & Coyle, 2020; Bell & Shine, 2016; Deco & Kringelbach, 2016). Dynamic reorganization of the network structure of the brain is directly linked to cognitive performance (Shine et al., 2016). Thus, dysfunction of subcortical regions in PD patients might contribute to abnormal functional integration and segregation of cortical networks. Investigating abnormal functional integration and segregation in PD and the underlying mechanism will provide a window for further understanding the complexities of cognitive deficits in PD.

Previous studies using resting‐state functional magnetic resonance imaging (fMRI) found alterations in functional connectivity that is related to motor and cognitive impairments (Gratton et al., 2019; Tahmasian et al., 2015). However, these studies report variable differences in functional connectivity, likely reflecting the heterogeneity of PD or differences in methodology (Gratton et al., 2019; Tahmasian et al., 2015). For example, both increased and decreased connectivity are often observed to be distributed among subcortical and cortical networks in the dopaminergic OFF state (O'Callaghan et al., 2016). Similarly, at the macroscopic level, both exaggeration of small‐world properties (Fiorenzato et al., 2019; Ko, Spetsieris, & Eidelberg, 2018) and an increase in network integration (Shine, Bell, et al., 2019) were observed in the dopaminergic OFF state. Currently, it is unclear which functional neuroimaging markers are reliable and convincing for understanding the consequences of PD rather than the mechanisms responsible for these pathological changes.

fMRI data are acquired as complex image pairs containing amplitude and phase components. However, the majority of fMRI studies do not utilize time‐dependent phase information, with the assumption that functional connectivity remains constant while the brain is in the resting state. Therefore, in the present study, we conducted a phase‐based synchronization analysis to measure the time‐dependent dynamic changes in functional interactions of the resting‐state fMRI signals. Recently, this approach has been successfully applied to fMRI studies on Alzheimer's disease patients and aging people, revealing a significant relationship between metastability and cognition (Alderson, Bokde, Kelso, Maguire, & Coyle, 2018; Deco & Kringelbach, 2016; Naik, Banerjee, Bapi, Deco, & Roy, 2017). Unlike functional correlation analysis, which is a linear measure of association between blood‐oxygen‐level‐dependent (BOLD) imaging signals, phase synchronization is a measure of statistical dependence that is sensitive to both linear and nonlinear relationships (Laird et al., 2002; Naik et al., 2017). By analyzing the instantaneous phases, time‐resolved nonlinear functional interactions are obtained at the same temporal resolution as the input fMRI signal. In this study, a phase‐based analysis was conducted using two measures: (a) synchronization, the average phase coupling between sets of resting‐state fMRI signals, and (b) metastability, phase‐based coupling, and decoupling over time, under the assumption that integration and segregation are reconciled (Alderson et al., 2018; Deco & Kringelbach, 2016; Naik et al., 2017). Synchronization is the degree of synchrony between oscillators in a network and metastability measures the variability in synchronization patterns at the network level from BOLD data (Alderson et al., 2020; Naik et al., 2017). A system with high metastability visits a range of different states over time (Alderson et al., 2020).

This study was aimed at investigating the potential implications of subcortical dysfunction on cortical synchronization/metastability and cognitive performance in PD patients. To examine the causal role of dopamine depletion in cortical synchronization and metastability, we also evaluated whether levodopa could modulate cortical synchronization and metastability in PD patients. Cortical synchronization and metastability were estimated by recording fMRI signals in the resting state across normal control (NC) individuals and PD patients both ON and OFF dopamine replacement therapy. We then tested whether cortical synchronization and metastability related to cognitive performance. Finally, we evaluated the impact of subcortical atrophy and reduction in subcortical–cortical structural connectivity on cortical synchronization and metastability.

## 2 MATERIALS AND METHODS

### 2.1 Participants and neuropsychological assessment

This study was approved by the Medical Ethics Committee of the Second Affiliated Hospital of Zhejiang University School of Medicine. All participants signed informed consent forms in accordance with the Declaration of Helsinki. We recruited 164 patients with idiopathic PD and 158 NC subjects from the Department of Neurology, Second Affiliated Hospital of Zhejiang University. PD patients were diagnosed by a senior neurologist according to UK Parkinson's Disease Brain Bank criteria. Participants were screened for a history of neurological or psychiatric disorders, and none were using psychoactive medications. Participants had no other neurological or major psychiatric illness, and their MRI scans presented no abnormal findings. In this study, we included 159 PD and 152 NC (five PD and six NC subjects were excluded because of head movement artifacts or cerebral hemorrhage (Figure S1). For patients receiving anti‐parkinsonian drugs, clinical assessments and MRI scans were performed during a drug‐free period (>12 hr, OFF state). Specifically, 75 patients were receiving levodopa monotherapy, 19 patients were on levodopa plus a dopaminergic agonist, 51 patients were on levodopa plus adjuvant therapy (selegiline, rasagiline, or entacapone), five patients were on dopaminergic agonist monotherapy, and nine patients were drug‐naïve. An equivalent dose of levodopa (LED) score was calculated for each patient.

Disease severity was assessed using the Unified Parkinson's Disease Rating Scale (UPDRS), Hoehn–Yahr (HY) stage, and the Parkinson's Disease Questionnaire (PDQ‐39, a disease‐specific health‐related quality of life instrument for patients with PD) (Peto, Jenkinson, & Fitzpatrick, 1998). Cognitive status was assessed using the Mini‐Mental State Examination (MMSE) and Montreal Cognitive Assessment (MoCA) (Nasreddine et al., 2005). All motor and cognitive assessments were conducted during the OFF state.

In addition, 45 of the 159 PD patients were evaluated clinically using the UPDRS‐part III (UPDRS‐III) in both OFF and ON states. An fMRI scan was also conducted during both of these periods. The OFF state is defined as 12 hr after the withholding of anti‐parkinsonian medication, and ON is defined as 1 hr after patients received a standard dose of dispersible levodopa–benserazide (200/50 mg). Specifically, 25 of these 45 patients were receiving levodopa monotherapy, three patients were on levodopa plus a dopaminergic agonist, and 17 patients were on levodopa plus adjuvant therapy (selegiline, rasagiline, or entacapone). The MMSE and MoCA were only assessed during the OFF state because of the memory effect.

### 2.2 Image acquisition

All participants in this study were scanned on a GE Discovery MR750 3.0 T MRI scanner equipped with an eight‐channel head coil. To reduce noise and head motion, earplugs and foam pads were used. High‐resolution 3D T1‐weighted structural imaging, diffusion tensor imaging (DTI), and fMRI data were acquired. Detailed scanning parameters are described in the supplementary materials.

### 2.3 Structural images processing

Gray matter (GM) extraction was performed using Computational Anatomy Toolbox 12 (http://www.neuro.uni-jena.de/cat/) running within Statistical Parametric Mapping 12 (www.fil.ion.ucl.ac.uk/spm). Preprocessing steps included denoising, correction for intensity inhomogeneity, and linear intensity scaling. Images were segmented into GM, white matter, and cerebrospinal fluid, and then normalized to the standard template (Montreal Neurological Institute [MNI] 152). A probabilistic segmentation approach was applied and a default threshold of 0.5 was used. The use of a group‐specific template (DARTEL) for spatial normalization on large sample sizes is prohibitively time‐consuming and was therefore not used. Then, prior to building the statistical model, the GM maps were smoothed using an 8‐mm full width at half‐maximum (FWHM) kernel. GM volumes were quantified in subcortical regions, including thalamus, putamen, caudate, and pallidum, which were defined using the Brainnetome Atlas (Fan et al., 2016). All subregions of each subcortical region were included. This atlas was selected because it has been validated in terms of both functional and structural anatomy and connectivity, consistent with the design and aims of the current study.

### 2.4 Resting‐state fMRI processing

Resting‐state fMRI preprocessing was performed using a MATLAB‐based package combining the FMRIB Software Library (FSL, https://fsl.fmrib.ox.ac.uk/fsl/fslwiki/) (Gregory, Long, Tabrizi, & Rees, 2017), the BrainWavelet toolbox (http://www.brainwavelet.org/) (Patel et al., 2014), and Analysis of Functional NeuroImages, (https://afni.nimh.nih.gov/) (Cox, 1996). The first 10 scans were discarded to suppress equilibration effects. The remaining scans of each subject underwent brain extraction, slice‐timing correction (FSL slicetimer), motion correction (FSL mcflirt), spatial smoothing with a 3D Gaussian kernel (FWHM = 6 mm), despiking of motion artifacts using the BrainWavelet Toolbox, band‐pass temporal filtering (0.01–0.1Hz, 3dTproject), and nuisance signal regression from the cerebrospinal fluid, white matter, and head motion using the 24‐parameter procedure. Participants with maximal motion between volumes in each direction >2 mm, and rotation about each axis >2° were excluded. Mean framewise displacements (Power et al., 2014) were also calculated (Figure S2) and were regressed out of all analyses. Finally, the processed images were registered to 3D T1 images and spatially normalized to a standard template (MNI) using FSL's linear and nonlinear registration tool (BBR, flirt, and fnirt in FSL). MATLAB implementations of the resting‐state fMRI preprocessing package are available on the GitHub page (https://github.com/weikanggong/Resting-state-fMRI-preprocessing).

### 2.5 DTI processing and structural connectome construction

DTI images were preprocessed using FSL (Jenkinson, Beckmann, Behrens, Woolrich, & Smith, 2012). The skulls were first stripped from the T1‐weighted images and DTI images for each participant. Then, eddy currents and head‐motion artifacts in diffusion data were corrected using eddy_correct in FSL (Andersson & Sotiropoulos, 2016). Finally, diffusion parameters (i.e., fractional anisotropy, mean diffusivity) were calculated using dtifit in FSL.

White matter tractography was reconstructed on the DTI using the MRtrix3 software package (http://www.mrtrix.org) (Tournier et al., 2019). The Brainnetome Atlas with 246 subcortical and cortical regions of interest was used to generate the nodes of the matrix. DTI tractography was performed to obtain internode white matter fiber tracts. The fiber assignment continuous tracking algorithm was applied to reconstruct internode white matter fiber tracts (streamline). Tractography was terminated if it turned at an angle >45° or reached a voxel with fractional anisotropy <0.2 (Guo et al., 2020). Prior to performing statistical analyses, fiber tracts between each seed and target node were divided by the mean fiber tracts of each subject (Mosley et al., 2019).

### 2.6 Calculating resting‐state network synchronization and metastability

Processed fMRI data were used for further phase analysis. The regional time series (BOLD signals) were extracted by averaging voxel‐level fMRI signals within each region of interest. For each participant, the regional time series (BOLD signals) $s_{j}\left(t\right)\;\left(j=1,2,\ldots ,210\right)$ were extracted. The Hilbert transform $H\left[s_{j}\left(t\right)\right]$ was applied to the BOLD signals to obtain the associated analytical signals with instantaneous phase traces $\theta_{j}\left(t\right)$:

$$\theta_{j}\left(t\right)=\arctan\frac{H\left[s_{j}\left(t\right)\right]}{s_{j}\left(t\right)}$$

**FIGURE 1:**
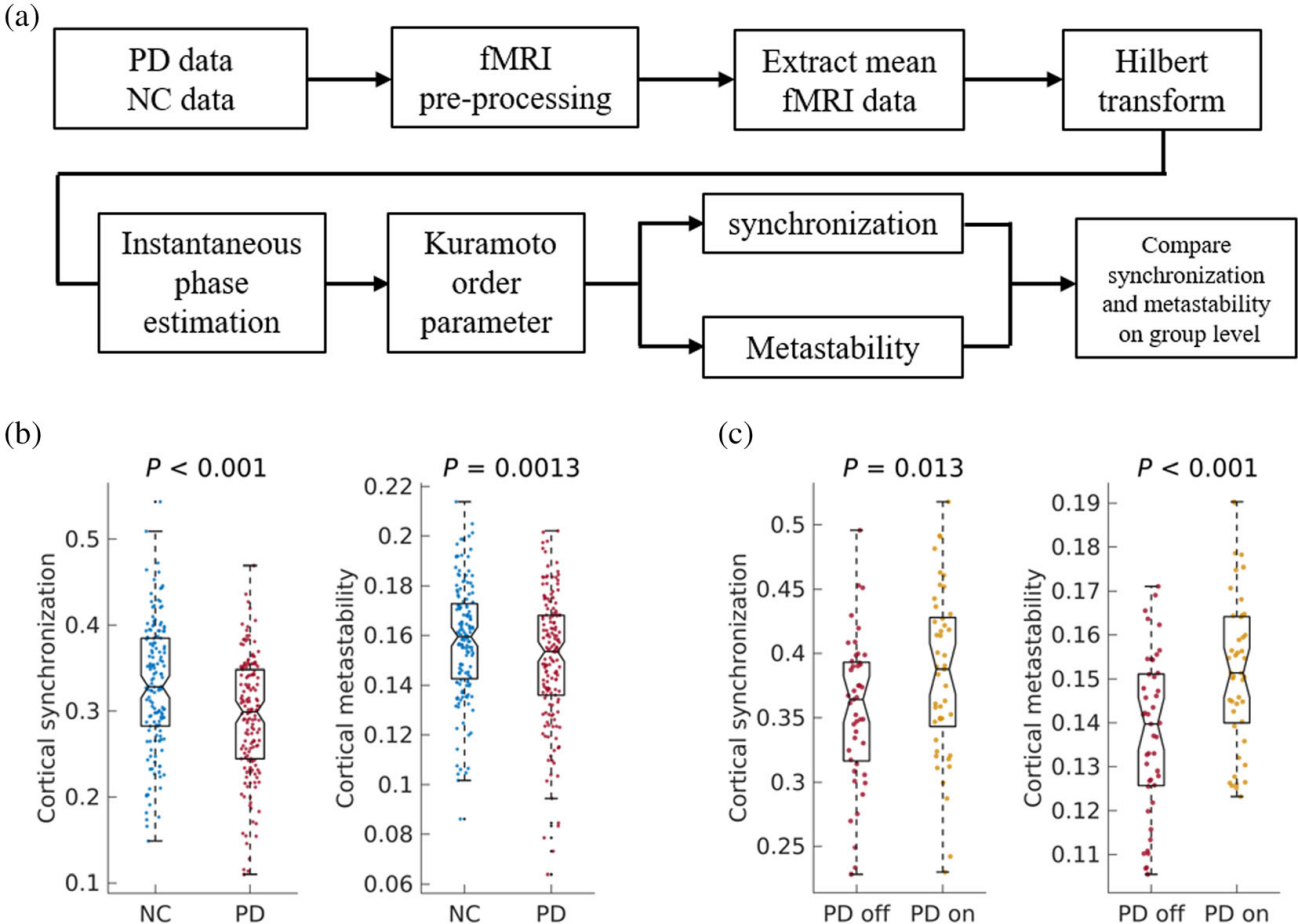
Workflow and results of the functional magnetic resonance imaging (fMRI) analysis. (a) An overview of the adopted fMRI analysis in this study. (b) Cortical synchronization and metastability in normal control (NC) individuals and Parkinson's disease (PD) patients. (c) Cortical synchronization and metastability in PD patients in the OFF and ON states. The values for OFF and ON states were compared using a paired‐sample *t* test after removing head motion

Then, we quantified the temporal averages of global‐level synchronization of *n* oscillating signals by the mean of the order parameter:

$$<r\left(t\right)>=\frac{1}{L}\sum_{t=1}^{L}r\left(t\right)$$

where *L* = 205 is the total time points of the BOLD signal and *r*(*t*) is the Kuramoto order parameter (Escaff & Delpiano, 2020), which is the canonical model for studying synchronization phenomena:

$$r\left(t\right)=\frac{1}{n}\left|\sum_{j=1}^{n}e^{i\theta_{j}\left(t\right)}\right|$$

and was used as a time‐dependent measure of phase synchrony. The metastability was then determined as the *SD* of *r*(*t*) (Alderson et al., 2018; Deco & Kringelbach, 2016; Wildie & Shanahan, 2012). Synchronization and metastability were estimated for (a) the set of 210 cortical regions and (b) the set of regions comprising single resting‐state networks (RSNs) including: visual, somatomotor, dorsal attention, ventral attention, limbic, and frontoparietal networks, and the default network derived from resting‐state fMRI by Yeo et al. (2011). These approaches were applied similarly to previous studies (Alderson et al., 2018; Demirtaş et al., 2016; Glerean, Salmi, Lahnakoski, Jääskeläinen, & Sams, 2012; Laird et al., 2002; Ponce‐Alvarez et al., 2015). A flow chart of the synchronization analysis is illustrated in Figure 1a.

### 2.7 Mediation analysis

Mediation analysis was performed using the mediation toolbox developed by Wager et al. (https://github.com/canlab/MediationToolbox) (Shrout & Bolger, 2002). Mediation analysis tests whether the covariance between two variables can be explained by a third variable (the mediator). A standard three‐variable path model was used here: path a—the effect of the independent variable on the mediator; path b—the effect of the mediator on the dependent variable; path c—the total effect of the independent variable on the dependent variable. Path c′—The direct effect of the independent variable on the dependent variable with the inclusion of the mediator; and path a × b—the effect of the independent variable on the dependent variable through the mediator. Age, gender, handedness, level of education, mean framewise displacements, and total intracranial volume were used as covariates of no interest. The significance of the mediation was estimated using the bias‐corrected bootstrap approach (with 10,000 bootstraps).

### 2.8 Statistical analysis

The relationship between parameters obtained by the imaging modalities listed and clinical scores was investigated using Pearson's correlation statistics. The *t* test was used to determine the statistical significance of continuous demographic and clinical variables. For the comparison of synchronization/metastability between OFF and ON states, paired *t* tests were conducted after regressing out mean framewise displacements. The χ^2^ test was used to test the significance of categorical demographic variables. For correlations between fiber tracts and clinical scores, principal component (PC) analysis was applied to the reconstructed fiber tracts that link subcortical regions and cortical regions to reorganize them into low‐dimensional PCs. Confounding factors such as age, gender, handedness, education level, total intracranial volume and mean framewise displacements were regressed out prior to statistical analysis. Statistical significance was established at *p* < .05, corrected for multiple comparisons (false discovery rate [FDR] correction).

## 3 RESULTS

### 3.1 Demographic and clinical characteristics

No significant difference was found between the NC and PD groups in gender or age. Education level was statistically different between the two groups, and thus was regressed out in the statistical analysis. PD patients had significantly lower MMSE and MoCA scores than the NC group. Detailed demographic and clinical characteristics are shown in Table 1.

**TABLE 1:** Demographic and clinical characteristics

	Normal controls (*n* = 152)	Parkinson's disease (*n* = 159)	*p*‐Value
Gender (male/female)	70/82	87/72	.13
Age (years)	60.9 ± 7.22	61.10 ± 9.53	.81
Education	9.66 ± 3.56	8.00 ± 4.54	*p* < .001
Duration (years)	—	3.90 ± 3.37	—
LED	—	410.23 ± 252.57	—
UPDRS‐III (OFF)	—	23.62 ± 12.66	—
HY stage	—	2.34 ± 0.53	—
MMSE	28.22 ± 1.66	27.01 ± 3.23	*p <* .001
MoCA	24.37 ± 3.46	22.09 ± 5.42 (*n* = 137)	*p <* .001
PDQ‐39	—	26.68 ± 20.16	—

*Note*: Only 137 patients had MoCA assessment.

Abbreviations: HY: Hoehn–Yahr; LED, equivalent dose of levodopa; MMSE: Mini‐Mental State Examination; MoCA, Montreal Cognitive Assessment; PDQ‐39:39‐item Parkinson's Disease Questionnaire; UPDRS‐III, Unified Parkinson's Disease Rating Scale part III.

The PD subgroup that underwent MRI scanning during the ON state (*n* = 45) were well matched in demographic and clinical characteristics with the whole PD group (*n* = 159). No significant differences were found in gender, age, education, duration, HY stage, and UPDRS‐III, PDQ‐39, MMSE, and MoCA scores between the two PD groups (Table S1).

There were no significant differences in head motion between NC individuals and PD patients in the OFF‐medication state (mean framewise displacements: NC: 0.12 ± 0.09, PD OFF: 0.12 ± 0.097, *p* = .40). Significantly less head motion was found in PD patients in the ON state than in the OFF state (mean framewise displacement: PD OFF: 0.16 ± 0.09, PD ON: 0.12 ± 0.08, *p* = .01).

### 3.2 Decrease of cortical synchronization and metastability in PD and its relationship with dopaminergic state

In the OFF state, PD patients showed lower levels of synchronization (the mean phase coherence over time) and simultaneously lower levels of metastability (the variation in the mean phase coherence over time) of the cortex, compared to the NC group (*p* < .05, FDR corrected; Figure 1b). The lower cortical synchronization suggests that PD patients exhibited less effective integration or a larger proportion of time spent in a segregation state compared to NC individuals. The lower cortical metastability suggests a less dynamic transition between global integration and a segregation state (Alderson et al., 2020). When patients were in an ON‐medication state, cortical synchronization and metastability were significantly greater than in the OFF state (*p* < .05, FDR corrected; Figure 1c), suggesting that dopamine depletion is partially responsible for the decrease of cortical synchronization and metastability in PD. When PD patients were receiving medication (ON state), cortical synchronization and metastability were still significantly lower in comparison to the NC group (*p* < .05, FDR corrected; Figure S3). These results indicated that the disrupted cortical synchronization and metastability were partially alleviated by levodopa.

### 3.3 Relationship between cortical synchronization and metastability and cognitive performance

**FIGURE 2:**
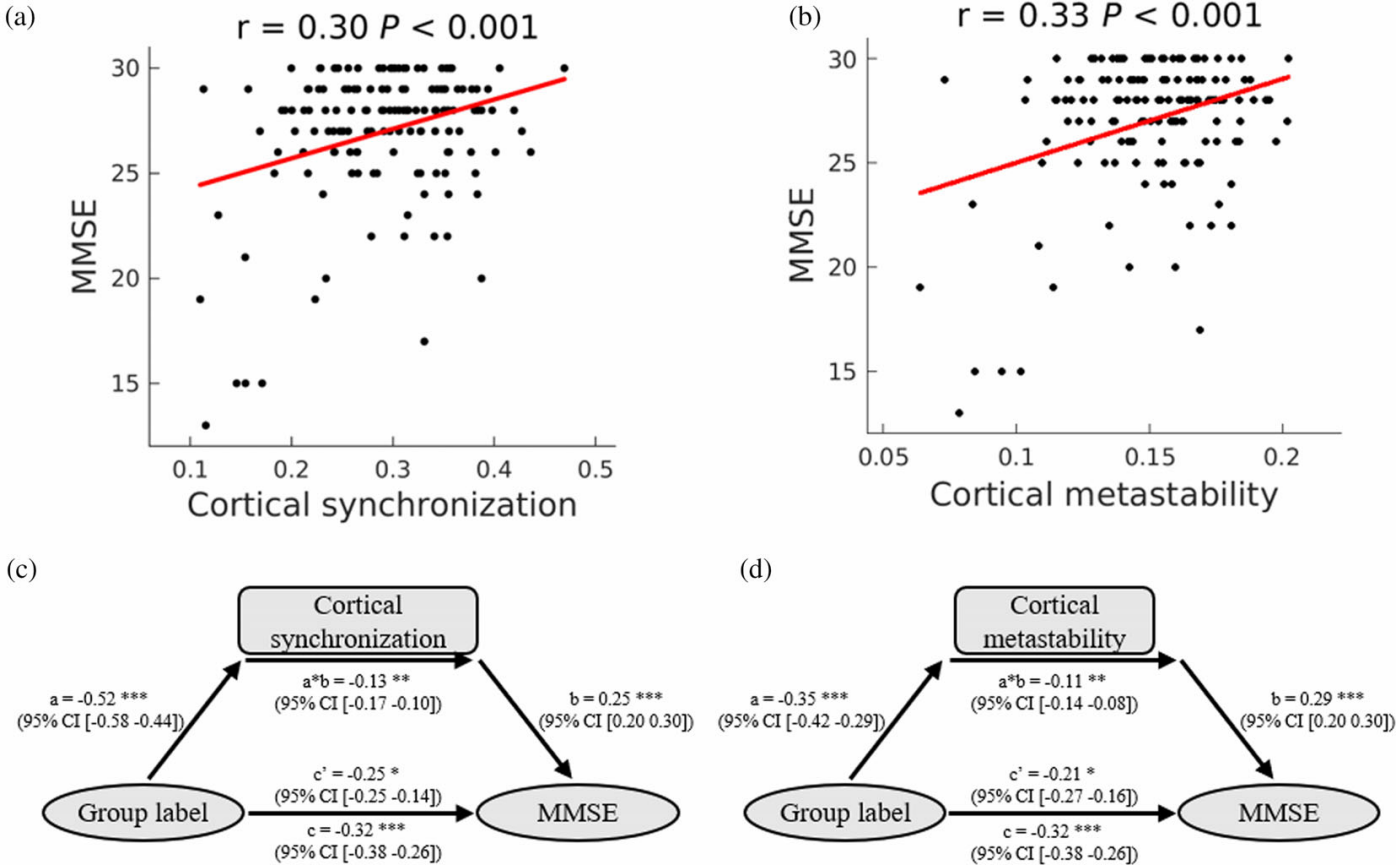
Relationship between cortical synchronization and metastability and cognitive performance. (a,b) Correlation analysis in Parkinson's disease (PD) patients. Pearson's correlation coefficients (*r*) and *p* values (false discovery rate [FDR] corrected) are shown. FDR correction was performed among four clinical scores. (a) Relationship between cortical synchronization and Mini‐Mental State Examination (MMSE) score. (b) Relationship between cortical metastability and MMSE score. (c,d) Mediation analysis. Mediation models using group label as the independent variable, cortical synchronization (c) and metastability (d) as the mediators, and MMSE score as the dependent variable. “Group labels” are the categorical labels of the normal control (NC) and PD groups, where NC was set as 0 and PD was set as 1. Path a measures the association between the predictor and the mediator; path b represents the effect of the mediator on the dependent variable; path c measures the total relationship between the predictor and the dependent variable; path c′ measures the effect of the predictor on the dependent variable while controlling for the mediator; the mediation effect is the product of paths a and b (a × b). *p < .05, **p < .01, ***p < .005

To test whether cortical synchronization and metastability are associated with cognitive performance, we tested for a correlation of cortical synchronization and metastability with MMSE and MoCA scores. We found both cortical synchronization and metastability positively correlated with MMSE (Figure 2a,b) and MoCA (Figure S4a,b) scores in the OFF state of PD patients (*p* < .05, FDR corrected), suggesting that lower cortical synchronization and metastability was associated with poorer cognitive performance. There was no such relationship in the NC group (*p* > .1), probably because their scores were closer to maximum. We then examined whether cortical synchronization and metastability mediated the difference of MMSE (Figure 2c,d) and MoCA (Figure S4c,d) scores between NC individuals and PD patients. The results show that the lower MMSE and MoCA scores in PD patients were significantly mediated by decreased cortical synchronization and metastability. Furthermore, only cortical metastability slightly correlated with UPDRS‐III score, but cortical synchronization did not (Figure S5a,b). Both cortical synchronization and metastability was negatively associated with PDQ‐39 score (Figure S5c,d). We conclude that worse mental health status and quality of life are associated with lower cortical synchronization and metastability.

### 3.4 Relationship between cortical synchronization and metastability and CBG architecture

The connections between CBG‐thalamus form part of a core circuit that supports large‐scale integration of information between distributed cortical regions (Oh et al., 2014); (Bell & Shine, 2016; Huo, Chen, & Guo, 2020). We therefore predicted a relationship between pathological change in CBG loop architecture and large‐scale communication within cortical regions.

**FIGURE 3:**
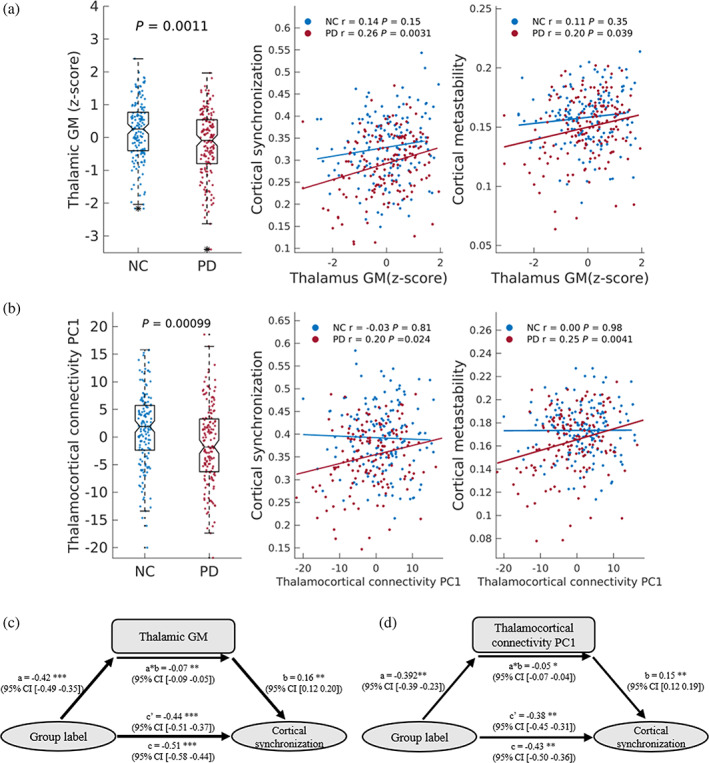
Relationships between cortical synchronization/metastability and thalamic gray matter volume and thalamocortical connectivity. (a,c) Relationships between cortical synchronization and metastability and thalamus gray matter volume (*n* = 159). (b,d) Relationships between cortical synchronization and metastability with thalamocortical connectivity (*n* = 152). Pearson's correlation coefficients (*r*) and *p* values (false discovery rate [FDR] corrected) are shown. FDR correction was performed among four subcortical regions. (c) Mediation model using group label as the independent variable, thalamus total gray matter volume as the mediator, and cortical synchronization as the dependent variable. (d) Mediation model using group label as the predictor, thalamocortical connectivity first principal component (PC1) as the mediator, and cortical synchronization as the dependent variable. “Group labels” are the categorical labels of normal control (NC) and Parkinson's disease (PD) groups, where NC was set as 0, PD was set as 1. *p < .05, **p < .01, ***p < .005

To test this hypothesis, we extracted GM volumes from the 3D T1 images. First, we tested whether there is a relationship between the overall GM volume and cortical synchronization and metastability. Of all the subcortical regions in the CBG architecture (i.e., thalamus, putamen, caudate, pallidum), we only observed positive correlations between the total GM volume of the thalamus and both cortical synchronization and metastability in PD patients (*p* < .05, FDR corrected; Figure 3a, Table S2). No correlation was found in the NC group. These results show that the greater the loss of thalamic GM, the greater the decrease in cortical synchronization and metastability. Then, we tested for significant differences in the volumes of the CBG architecture between PD patients and NC individuals. The results revealed significant differences in the thalamus, putamen, and pallidum (*p* < .05, FDR corrected; Figure 3a, Table S3). We then examined whether these differences in GM volume mediated the decrease of cortical synchronization and metastability in PD patients. The results show that the decrease of cortical synchronization in PD patients was significantly mediated by the loss of GM in the thalamus (Figure 3c).

Next, we tested whether cortical synchronization and metastability were associated with fiber connectivity in the CBG loop. A PC analysis was applied to the reconstructed fiber tracts that link subcortical regions and cortical regions to reorganize them into low‐dimensional PCs. The first PC (PC1) of thalamocortical connectivity is positively correlated with cortical synchronization and metastability in the PD group (*p* < .05, FDR corrected; Figure 3b and Table S3). The PC1 show significant differences between PD patients and NC individuals. The decrease of cortical synchronization in PD patients was also significantly mediated by the PC1 of thalamocortical connectivity (Figure 3d).

### 3.5 Relation between cognition and thalamus dysfunction

**FIGURE 4:**
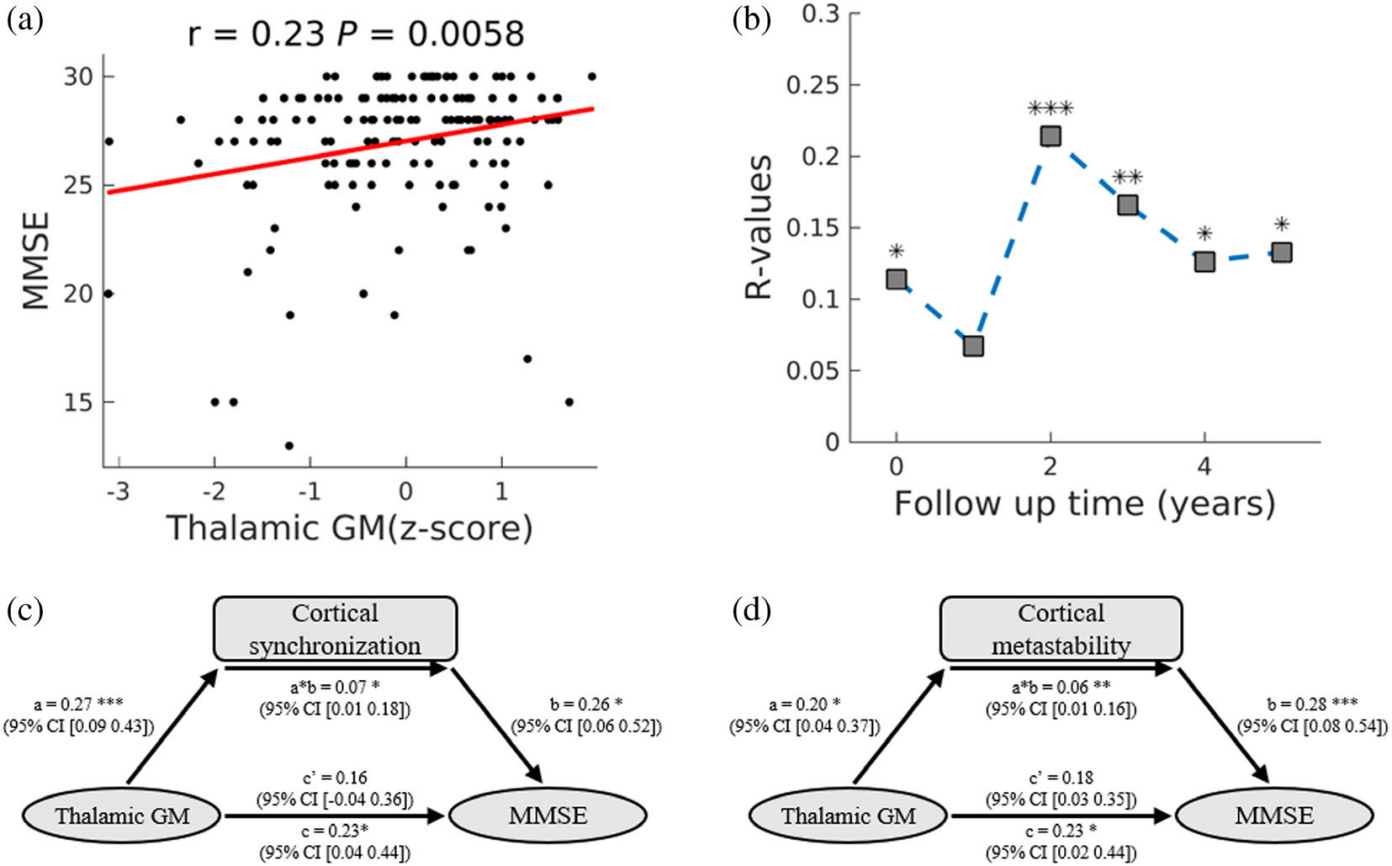
Correlation between thalamus gray matter volume and cognitive score. Pearson's correlation coefficients (*r*) and *p* values (false discovery rate [FDR] corrected) are shown. (a) Relationship between thalamus gray matter volume and Mini‐Mental State Examination (MMSE) score. (b) The correlation between thalamus gray matter volume and Montreal Cognitive Assessment (MoCA) score at both baseline and follow‐up visits using data from the PPMI dataset. (c,d) Mediation analysis. Mediation model using thalamus gray matter volume as the independent variable, cortical synchronization (c) and metastability (d) as the mediator, and cortical synchronization as the dependent variable

As both thalamic GM volume and the PC1 of thalamocortical connectivity positively correlated with cortical synchronization and metastability, we tested whether these measures also correlated with cognitive score. The results show that thalamic GM volume positively correlated with MMSE and MoCA (*p* < .05, FDR corrected; Figure 4a, Figure S6a,b) scores in the OFF state. The correlation between PC1 of thalamocortical connectivity and MMSE score (*p* = .04, uncorrected) not survived correction for multiple comparisons (Figure S6c,d). We also used a separate data set—the Parkinson's Progression Markers Initiative (PPMI) (Marek et al., 2018)—to test whether the relationship between cognition and thalamic GM is robust. PD participants enrolled in this dataset receiving visits for up to 5 years. The demographic and MoCA of these patients are provided in Table S4. We observed significant associations between baseline thalamic GM and MoCA score assessed at both baseline and four follow‐up visits (2–5 years; Figure 4b, Figure S7). These results suggest that thalamus atrophy has an effect on cognitive performance.

Next, we investigated the extent to which the relationship between brain structure and cognition was mediated by cortical synchronization and metastability. The result shows that the relationship between thalamic GM volume and cognitive score (MMSE and MoCA) was significantly mediated by cortical synchronization and metastability (Figure 4c,d, Figure S6e,f). These results, combined with those shown in Figure 3, provide evidences that the differences in CBG loop structure described here and cortical synchronization and metastability are closely related to cognitive performance.

### 3.6 Decrease in synchronization and metastability of RSNs and subcortical–cortical connectivity

**FIGURE 5:**
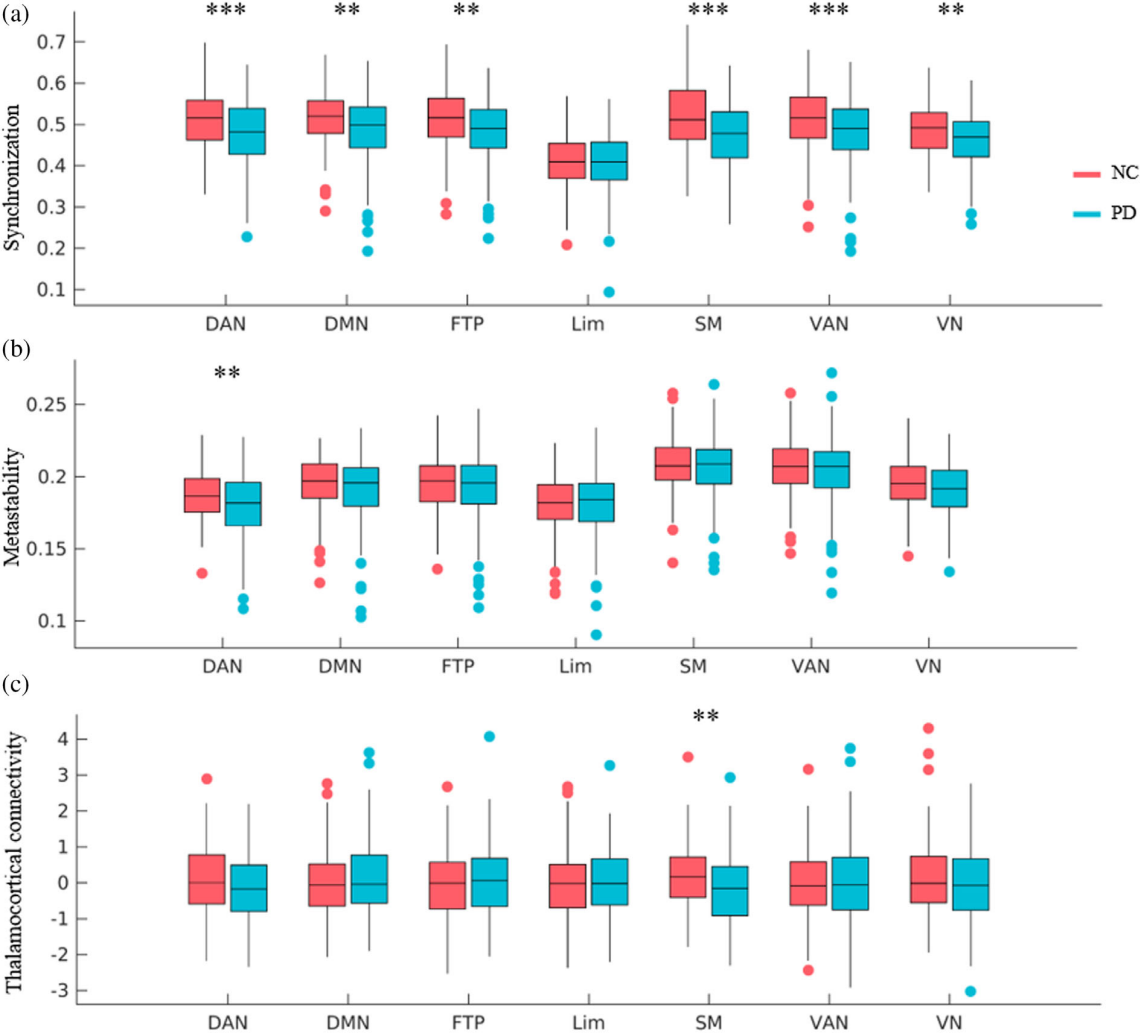
Relationships between network synchronization and disruption of subcortical–cortical structural connectivity. (a) Synchronization analyses of blood‐oxygen‐level‐dependent (BOLD) data within each of the seven resting‐state networks (RSNs) delineated by Yeo et al. (b) Metastability analyses of BOLD data within each of the seven RSNs. (c) Thalamocortical connectivity (average of connectivity between the thalamus and regions in each network) in Parkinson's disease (PD) patients versus normal control (NC) individuals in the seven RSNs. False discovery rate (FDR) correction was performed among seven within‐network. DAN, dorsal attention network; DMN, default mode network; FTP, frontoparietal network; Lim, limbic network; SMN, somatomotor network; VAN, ventral attention network; VN, visual network. *p < .05, **p < .01, ***p < .005, FDR corrected

Previous studies have shown that PD patients with cognitive deficits have increased local connectivity (exaggeration of the small‐world property) and decreased long‐range connectivity (Baggio et al., 2014; Fiorenzato et al., 2019; Ko et al., 2018). In order to test whether synchronization and metastability of RSNs were also increased, we assigned each cortical parcel to one of the seven RSNs derived from resting‐state fMRI by Yeo et al. (2011) and estimated their synchronization and metastability. Decreases of synchronization were observed across multiple RSNs, including visual, somatomotor, dorsal attention, ventral attention, frontoparietal, default, but not in limbic (*p* < .05, FDR corrected; Figure 5a). Overall, these changes suggested that in PD patients, not only were global interactions significantly decreased, but within‐network interactions were also proportionally decreased. However, significant decrease of metastability was only observed for the dorsal attention network in PD patients (*p* < .05, FDR corrected; Figure 5b). In addition, we also observed numerous correlations between the synchronization and metastability of the networks and disease severity (*p* < .05, FDR corrected; Figure S8), confirming the relationship between PD symptom severity and synchronization in the OFF state. Notably, the synchronization of the somatomotor network correlated with UPDRS‐III, suggesting that motor dysfunctions associated with lower synchronization of somatomotor network. In addition, when patients were in the ON state, the synchronization of somatomotor, visual, and dorsal attention networks and the metastability of dorsal attention, frontoparietal networks were significantly improved (*p* < .05, FDR corrected; Figure S9).

Growing evidence suggests that a “prion‐like” mechanism underlies the pathogenesis of PD (Pandya et al., 2019). To further determine the relationships between decreased synchronization and structural disruption, we assessed the alterations in structural connectivity between subcortical regions and each RSN. Structural connectivity linking the subcortical regions (i.e., thalamus, putamen, caudate, and pallidum) to somatomotor network was, on average, significantly impaired in PD patients compared to NC individuals (Figure S10a–c). Furthermore, structural connectivity linking the caudate and pallidum to the dorsal attention network was also, on average, significantly impaired in PD patients (Figure S10b,c). Considering that the subcortical areas comprise a putative “disease reservoir” in PD (Pandya et al., 2019; Yau et al., 2018), these results suggest that disease propagation to the cortex not only follows but also disrupts neuronal connectivity.

## 4 DISCUSSION

In this study, we found a simultaneous decrease of cortical synchronization and metastability in PD patients that was related to cognitive performance. Cortical synchronization and metastability mediated cognitive impairment of PD patients, relative to NC individuals. Moreover, we found a causal role for dopamine depletion in the decrease of cortical synchronization and metastability in PD patients. Furthermore, the decrease of cortical synchronization and metastability was also related to thalamic atrophy. Finally, thalamic volume correlated with cognitive performance, and this relationship was mediated by cortical synchronization and metastability. Overall, our findings suggest a key linkage between dopamine depletion/thalamic dysfunction and the decrease of cortical synchronization and metastability and cognitive impairment in patients with PD.

Besides cognitive scores (assessed by MMSE and MoCA), we also found cortical synchronization/metastability correlated with quality of life (as assessed by PDQ‐39), which was consistent with previous findings that cognitive impairment had a great impact on quality of life in PD patients (Tang et al., 2020). We also found that the synchronization of the somatomotor network correlated with motor dysfunction (assessed by UPDRS‐III), but the correlation between cortical synchronization/metastability and motor dysfunction was not significant, corroborating the independence of the motor‐associated property. The MMSE, MoCA, and PDQ‐39 assessments measure the severity of PD by testing an individual's abilities related to language, attention, memory, emotions and motor function. All these domains involve the large‐scale integration of information across distributed sensory, emotion, and cognitive control regions. Levodopa treatment significantly increased cortical synchronization and metastability in PD patients, suggesting that a decrease was partially caused by dopamine deficiency. Although we did not conduct cognitive assessments after levodopa treatment because of the memory effect, previous studies have reported dopamine modulation improves cognitive performance in both PD patients (Spalletta et al., 2014; Tahmasian et al., 2015) and healthy individuals by boosting working memory as well as visuospatial and attentional processing (Westbrook & van den Bosch, 2020).

Although previous studies on functional connectivity have found a link between dynamic connectivity and cognitive performance in PD (Fiorenzato et al., 2019; Kim et al., 2017; Shine et al., 2016), the structural basis underlying this is largely unknown. In this study, we found associations between cortical synchronization/metastability and pathological changes in thalamic volume and thalamocortical fiber connectivity in PD patients. A mediation analysis further demonstrated that these pathological changes significantly mediated the differences in cortical synchronization and metastability between PD and NC groups. This is consistent with previous studies showing that the higher‐order thalamus regulates the synchrony between cortical neurons and, consequently, cognitive processing (Saalmann, 2014), and that thalamus shape is significantly associated with cognitive decline in PD patients (Chung et al., 2017; Filippi et al., 2020). Previously, we also found that PD patients with lower subcortical brain volume declined more rapidly in several clinical domains, for example, cognition (Wang, Cheng, & Rolls, 2020). Moreover, the relationship between thalamic volume and MoCA score revealed by our dataset was confirmed with the PPMI dataset. Therefore, we postulate that structural and functional changes within subcortical structures contribute to diminished dynamic integration and segregation of large‐scale cortical networks, which leads to cognitive impairment (Jones, 2001; Saalmann, 2014). Our collective findings provide important insights into the mechanistic underpinnings of cognitive impairment in PD, which might help to predict the risk of cognitive decline over time. Currently, the state of the art in determining an individual's risk of dementia in PD is clinical–genetic algorithms (Liu et al., 2017; Schrag, Siddiqui, Anastasiou, Weintraub, & Schott, 2017). Future work combining demographic data, clinical scores, genetic analysis, as well as studies on thalamic GM and cortical synchronization and metastability, will enable more robust identification of at‐risk patients before dementia has taken hold.

The propagation model of PD predicts that α‐synuclein deposition progresses from the brainstem to subcortical nuclei, and then affects cortical regions, which in turn results in neuronal death (Pandya et al., 2019; Yau et al., 2018). The decrease of synchronization in the somatomotor network is the most prominent among the seven networks and is associated with UPDRS‐III score. Consistent with this, the most prominent RSN disruption and subcortical–cortical structural connectivity in our PD sample is the connectivity between subcortical nuclei and somatomotor networks. Accordingly, we speculate that somatomotor regions as well as fibers linking with subcortical regions are the first of the brain networks affected by α‐synuclein deposition (Campbell et al., 2015).

Finally, the potential limitations of this study need to be acknowledged. First, only a small sample of PD patients was scanned in both ON and OFF states of dopamine replacement therapy. Future studies could include a greater number of patients scanned in both states. Considering that this was a single‐center, cross‐sectional study, the current findings should be externally validated in an independent database in future studies. Cognition was evaluated using MMSE and MoCA assessing general cognitive function (Zadikoff et al., 2008), which does not meet the level II criteria of cognitive assessments (Emre et al., 2007; Goldman & Holden, 2018). Cognitive status was not assessed during the ON state. A more comprehensive evaluation of the relationship between dopaminergic state and cognitive performance in PD patients is required in the future. Meanwhile, we acknowledged that 12 hr may not enough for drug washout in patients with dopamine agonists or monoamine oxidase inhibitor.

## 5 CONCLUSION

This study demonstrated that a combination of dopamine depletion and the dysfunction of subcortical‐related structures leads to disruptions in cortical synchronization and metastability, which might help explain cognitive impairment in PD.

## CONFLICT OF INTEREST

The authors declare no potential conflict of interests.

## AUTHOR CONTRIBUTIONS

**Linbo Wang** and **Cheng Zhou**: Research project‐conception and execution, statistical analysis‐design and execution, manuscript‐writing of the first draft. **Wei Cheng**, **Edmund T. Rolls**, and **Peiyu Huang**: Statistical analysis‐review, manuscript‐review. **Ningning Ma**, **Yuchen Liu**, and **Yajuan Zhang**: Data‐preprocessing. **Xiaoujun Guan**, **Tao Guo**, **Jingjing Wu**, **Ting Gao**, **Min Xuan**, **Quanquan Gu**, **Xiaojun Xu**, and **Bao‐Rong Zhang**: Data‐collection. **Weikang Gong**, **Jingnan Du**, and **Wei Zhang**: Data‐preprocessing. **Jianfeng Feng** and **Minming Zhang**: Research project‐conception and organization; manuscript‐review.

## ETHICS STATEMENT

This study was approved by the Medical Ethics Committee of the Second Affiliated Hospital of Zhejiang University School of Medicine.

## PATIENT CONSENT STATEMENT

All participants signed informed consent forms in accordance with the Declaration of Helsinki.

## Supporting information


**Appendix** **S1**: Supporting Information

## Data Availability

The datasets generated and/or analyzed during the current study are available from the corresponding author on reasonable request.
