# Supplementary material for: Dopamine depletion and subcortical dysfunction disrupt cortical synchronization and metastability affecting cognitive function in Parkinson's disease
Source: Hum Brain Mapp. 2021 Dec 14;43(5):1598–610. doi: 10.1002/hbm.25745 (PMC8886656; doi:10.1002/hbm.25745)

# Supplementary materials

## Image acquisition and preprocessing

High resolution 3D T1 weighted structural imaging data were acquired using a fast-spoiled gradient recalled echo sequence: echo time (TE) = 3.036 ms; repetition time (TR) = 7.336 ms; inversion time = 450 ms; flip angle (FA) = 11 degrees; field of view (FOV) = 260 × 260 mm^2^; matrix = 256 × 256; slice thickness = 1.2 mm; number of slices = 196 (sagittal).

Acquisition parameters for fMRI data were as follows: TE = 30 ms; TR = 2,000 ms; FA = 77 degrees; FOV = 240 × 240 mm^2^; matrix = 64 × 64; slice thickness = 4 mm; slice gap = 0 mm; number of slices = 38 (axial).

Diffusion Tensor Imaging (DTI) data were acquired using the following parameters: TR = 8000 ms; TE = 80 ms; flip angle = 90 degrees; field of view = 256 × 256 mm^2^; matrix = 128 × 128; slice thickness = 2 mm; voxel size = 2×2 ×2, slice gap = 0 mm; and 67 interleaved axial slices. Diffusion images were acquired from 30 gradient directions (b = 1000 s/mm2), and included five acquisitions without diffusion weighting (b = 0).

## fMRI data preprocessing

**Preprocessing Steps:**

a) fMRI data brain extraction (bet in FSL).

b) Slice timing correction (slicetimer in FSL).

c) Motion correction (mcflirt in FSL).

d) Smoothing (fslmath in FSL).

e) Wavelet Despiking (BrainWavelet toolbox).

f) Regress out WM/CSF/Motion parameters and temporal filtering (3dTproject in AFNI).

g) FunImg register to T1, then normalize to 3mm Standard Space (BBR, flirt and fnirt in FSL).

**Despiking output:**

The mean corrected spikes were 36 time points for healthy control group, 34 for PD group (OFF state), and 28 for PD-ON state.

**Participants from PPMI cohort**

The PPMI is a prospective, longitudinal, observational multicenter study that aims to verify biomarkers of PD. Cognitive assessments for participants from the PPMI occurred at baseline and during the subsequent 4 years. Gray matter (GM) extraction was performed using Computational Anatomy Toolbox 12. Raw images of lower quality (CAT image quality rating <75%) were excluded. Therefore, 364 PD patients were included from the PPMI cohort. Clinical and demographic details for the PPMI are provided in the Supplementary Data (Supplementary Table 2). The study was approved by the institutional review board at each PPMI site. Written informed consent was obtained from all patients participating in the study.

**A MATLAB script that used for calculating resting state network synchronization and metastability**

*function [synchronization, metastability] = phase_synchronization(brainnetome_rsfMRI_2D)*

*% Phase synchrony analysis for estimating synchronization and metastability:*

*% Input: rsfMRI_2D, 2D fMRI data (time-points x nodes, or T x N).*

*% Output: synchronization and metastability.*

*id_num = length(brainnetome_rsfMRI_2D);*

*time =size(brainnetome_rsfMRI_2D{1},1);*

*region= size(brainnetome_rsfMRI_2D{1},2);*

*synchronization = zeros(id_num,1);*

*metastability = zeros(id_num,1);*

*for n = 1:id_num*

*time_series = brainnetome_rsfMRI_2D{n};*

*H = zeros(time, region);*

*Theta = zeros(time, region);*

*r = zeros(time,1);*

*for j = 1:region*

*H(:,j) = hilbert(time_series(:,j));*

*end*

*for k = 1:time*

*for j = 1:region*

*Theta(k,j) = angle(H(k,j));*

*end*

*r(k) = abs(sum(exp(1i*Theta(k,:))))/region;*

*end*

*synchronization(n) = mean(r);*

*metastability(n) = std(r);*

*end*

*end*

**Table S1. Sample homogeneity test between PD groups at OFF and ON states.**

|  | **Parkinson’s disease**  **(159)** | **Parkinson’s disease**  **(45)** | **P-value** |
| --- | --- | --- | --- |
| Gender (male/female) | 87/72 | 28/17 | 0.308 |
| Age | 60.99±9.62 | 59.44±8.64 | 0.295 |
| Education | 7.96±3.79 | 8.4±4.25 | 0.560 |
| Duration (year) | 4.09±3.79 | 4.37±3.51 | 0.514 |
| LED | 410.23±252.57 | 398.19±133.01 | 0.759 |
| UPDRS-III (OFF) | 23.8±12.65 | 21.93±15.04 | 0.415 |
| UPDRS-III (ON) | - | 14.54±12.19 | - |
| HY stage | 2.34±0.52 | 2.29±0.73 | 0.907 |
| MMSE | 26.89±3.51 | 27±3.78 | 0.819 |
| MoCA | 22.09 ± 5.42 (*n = 137*) | 22.61±5.38 | 0.670 |
| PDQ-39 | 26.94±20.50 | 21.29±16.09 | 0.059 |

LED: Equivalent dose of levodopa; UPDRS: Unified Parkinson’s Disease Rating Scale; HY: Hoehn-Yahr; MMSE: Mini-mental State Examination; MoCA: Montreal Cognitive Assessment; PDQ-39: the Parkinson's Disease Questionnaire (39 items).

# Table S2. Relationships between cortical synchronization/metastability and subcortical gray matter.

| **Subcortical GM of PD and NC** | | | | | | |
| --- | --- | --- | --- | --- | --- | --- |
|  | **PD_mean** | | **NC_mean** | **T** | ***P*** | ***P* (FDR corrected)** |
| Thalamus | 1940.06±275.6 | | 2047.80±245.54 | -3.678 | <0.001 | 0.001 |
| Caudate | 502.32±184.6 | | 542.37±174.95 | -1.986 | 0.048 | 0.064 |
| Putamen | 992.09±278.23 | | 1044.20±259.6 | -1.726 | 0.085 | 0.085 |
| Pallidus | 181.53±51.26 | | 197.20±55.31 | -2.623 | 0.009 | 0.018 |
| **Relationships between cortical synchronization and subcortical gray matter** | | | | | | |
|  | **NC** | | | **PD** | | |
|  | R | *P* | *P* (FDR corrected) | R | *P* | *P* (FDR corrected) |
| Thalamus | 0.137 | 0.092 | 0.149 | 0.264 | 0.001 | 0.003 |
| Caudate | 0.13 | 0.111 | 0.149 | 0.057 | 0.475 | 0.917 |
| Putamen | 0.138 | 0.091 | 0.149 | 0.008 | 0.917 | 0.917 |
| Pallidus | 0.016 | 0.848 | 0.848 | -0.029 | 0.714 | 0.917 |
| **Relationships between cortical metastability and subcortical gray matter** | | | | | | |
|  | **NC** | | | **PD** | | |
|  | R | *P* | *P* (FDR corrected) | R | *P* | *P* (FDR corrected) |
| Thalamus | 0.111 | 0.172 | 0.351 | 0.204 | 0.01 | 0.039 |
| Caudate | 0.11 | 0.176 | 0.351 | 0.144 | 0.07 | 0.14 |
| Putamen | 0.088 | 0.281 | 0.374 | 0.097 | 0.225 | 0.299 |
| Pallidus | -0.035 | 0.671 | 0.671 | 0.042 | 0.602 | 0.602 |

NC: Normal controls; PD: Parkinson’s disease.

# Table S3. Relationships between cortical synchronization/metastability and Subcortical-cortical connectivity.

| **Subcortical-cortical connectivity of PD and NC** | | | | | | |
| --- | --- | --- | --- | --- | --- | --- |
|  | **PD_mean** | | **NC_mean** | **T** | ***P*** | ***P* (FDR corrected)** |
| Thalamus | -1.38±7.20 | | 1.38±6.82 | 3.409 | 0.001 | 0.001 |
| Caudate | 0.21±3.67 | | -0.21±3.68 | -0.967 | 0.334 | 0.334 |
| Putamen | -0.73±2.49 | | 0.73±2.73 | 4.853 | <0.001 | <0.001 |
| Pallidus | 0.71±2.65 | | -0.71±2.66 | -4.656 | <0.001 | <0.001 |
| **Relationships between cortical synchronization and Subcortical-cortical connectivity** | | | | | | |
|  | **NC** | | | **PD** | | |
|  | R | *P* | *P* (FDR corrected) | R | *P* | *P* (FDR corrected) |
| Thalamus | -0.031 | 0.704 | 0.811 | 0.205 | 0.012 | 0.024 |
| Caudate | 0.138 | 0.091 | 0.362 | -0.142 | 0.082 | 0.11 |
| Putamen | 0.02 | 0.811 | 0.811 | 0.064 | 0.436 | 0.436 |
| Pallidus | -0.063 | 0.444 | 0.811 | -0.26 | 0.001 | 0.005 |
| **Relationships between cortical metastability and Subcortical-cortical connectivity** | | | | | | |
|  | **NC** | | | **PD** | | |
|  | R | *P* | *P* (FDR corrected) | R | *P* | *P* (FDR corrected) |
| Thalamus | 0.004 | 0.96 | 0.981 | 0.249 | 0.002 | 0.004 |
| Caudate | 0.057 | 0.484 | 0.981 | -0.108 | 0.185 | 0.247 |
| Putamen | 0.042 | 0.607 | 0.981 | 0.094 | 0.25 | 0.25 |
| Pallidus | 0.002 | 0.981 | 0.981 | -0.274 | 0.001 | 0.003 |

NC: Normal controls; PD: Parkinson’s disease.

**Table S4. Demographic and clinical characteristics of PD patients in PPMI**.

|  | **Parkinson’s disease**  **(*n* = 364)** |
| --- | --- |
| Gender (male/female) | 233/131 |
| Age (years) | 61.42 ±9.93 |
| Education | 15.57 ± 2.99 |
| Duration (year) | 0.57 ± 0.56 |
| MoCA (Baseline) | 27.18 ± 2.34 |
| MoCA (V04) | 26.4 ± 2.82 |
| MoCA (V06) | 26.3 ± 3.16 |
| MoCA (V08) | 26.49 ± 2.95 |
| MoCA (V10) | 26.46 ± 3.54 |
| MoCA (V12) | 26.58 ± 3.59 |

MoCA: Montreal Cognitive Assessment.

# Supplementary Figures legends

# Figure S1. Participant enrollment.


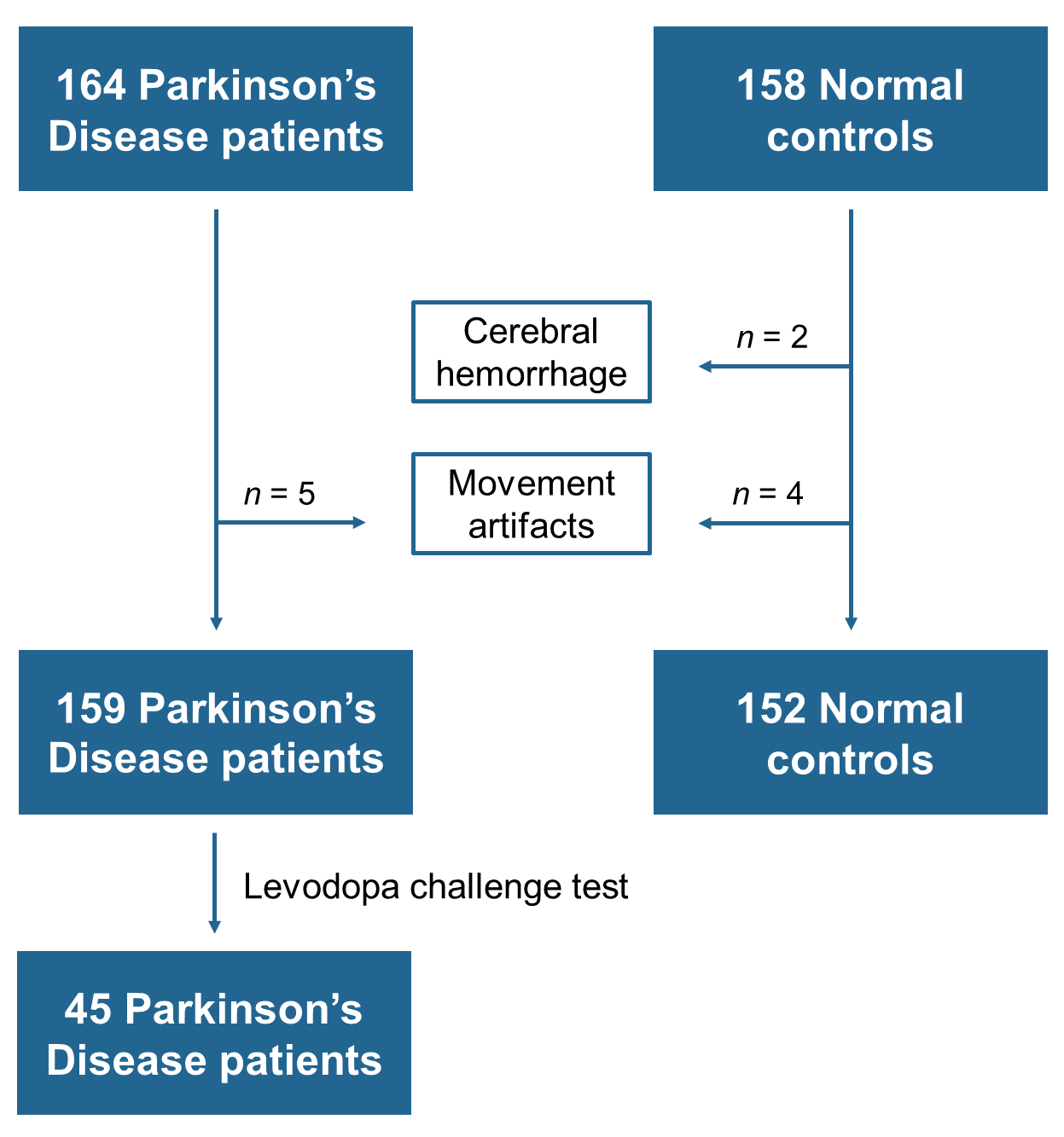


# Figure S2. The mean framewise displacement of all participants. NC: Normal controls; PD OFF: Parkinson’s disease during OFF state; PD ON: Parkinson’s disease during ON state.

# Figure S3. Cortical synchronization and metastability difference between Parkinson’s disease patients during ON state (PD on) and normal controls (NC).


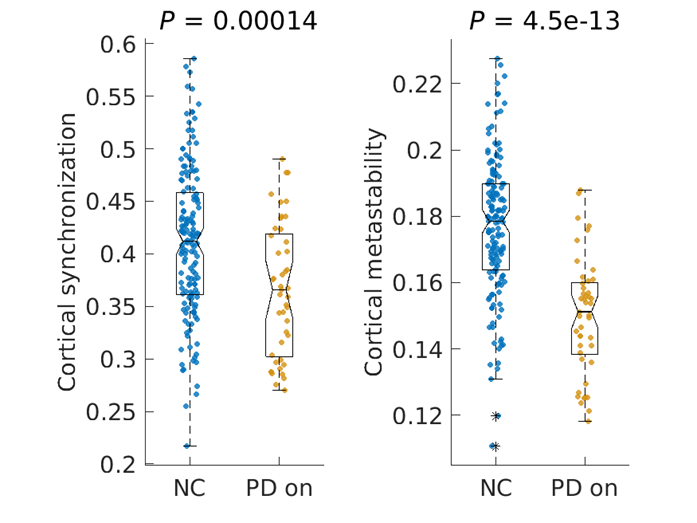


**Figure S4. Relationship between cortical synchronization and metastability and MoCA score.** (A, B) Correlation analysis. r values (Pearson’s correlation coefficient) and *P*-values (FDR corrected) are provided. (A) Relationship between cortical synchronization and MoCA score. (B) Relationship between cortical metastability and MoCA score. (C, D) Mediation analysis. Mediation model using group label as the independent variable, cortical synchronization (C) and metastability (D) as the mediators, and MoCA score as the dependent variable. Group labels are categorical label of normal controls (NC) and Parkinson’s disease patients (PD), where NC were set as 0, PD were set as 1. Age, sex, handedness, education level, head motion, and total intracranial volume were used as covariates of no interest. * *p* < 0.05, ** *p* <0.01, *** *p* < 0.005.


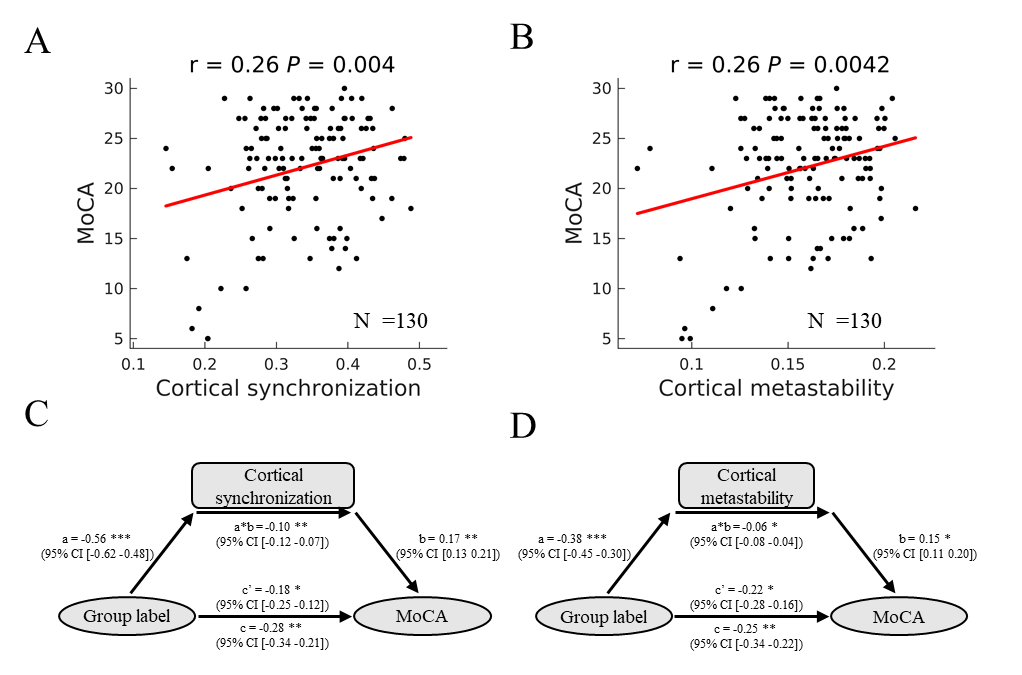


# Figure S5. Correlation between cortical synchronization and metastability with clinical scores. r values (Pearson’s correlation coefficient) and p values (FDR corrected) are provided. (A, C) Correlation between cortical synchronization and UPDRS-III (A) and PDQ-39 (C). (B, D) Correlation between cortical metastability and UPDRS-III (B) and PDQ-39 (D).


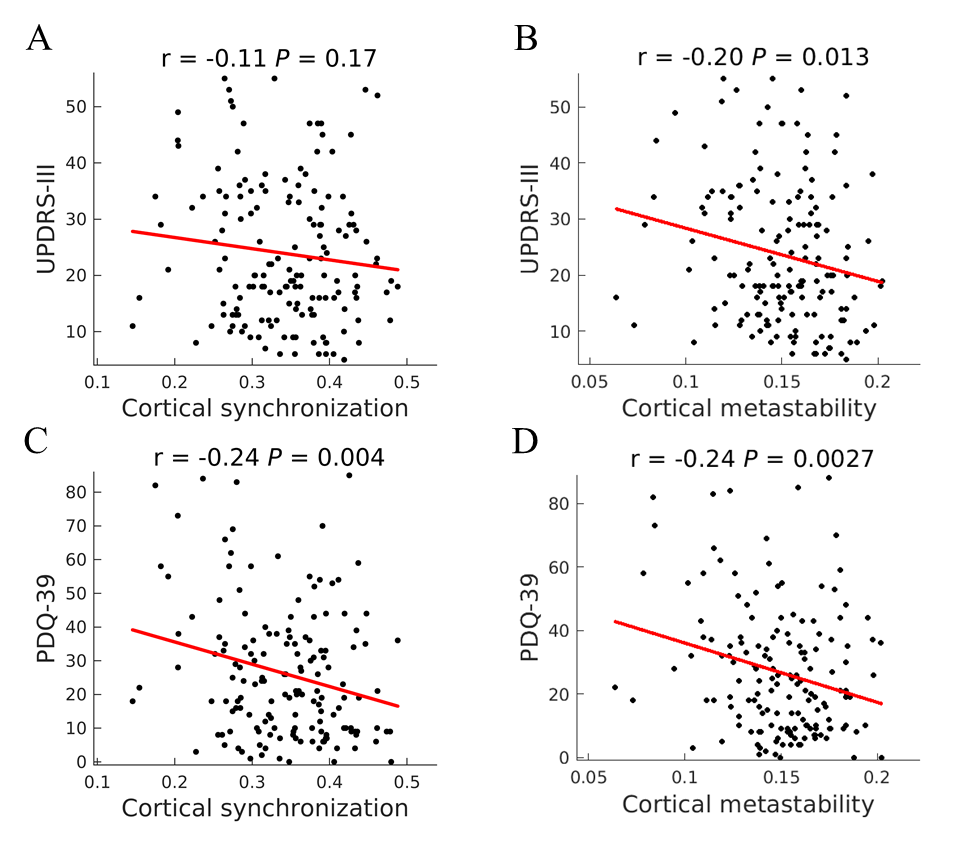


# Figure S6. Relationship between thalamic GM volume and PC1 of thalamocortical connectivity and MoCA score. (A, B) Relationship between thalamic GM volume and MMSE (A), MoCA (B) in PD group. (C, D) Relationship between PC1 of thalamocortical connectivity and MMSE (C), MoCA (D) in PD group. (E, F) Mediation analysis. Mediation model using thalamus gray matter volume as the independent variable, cortical synchronization (E) and metastability (F) as the mediator, and MoCA as the dependent variable.


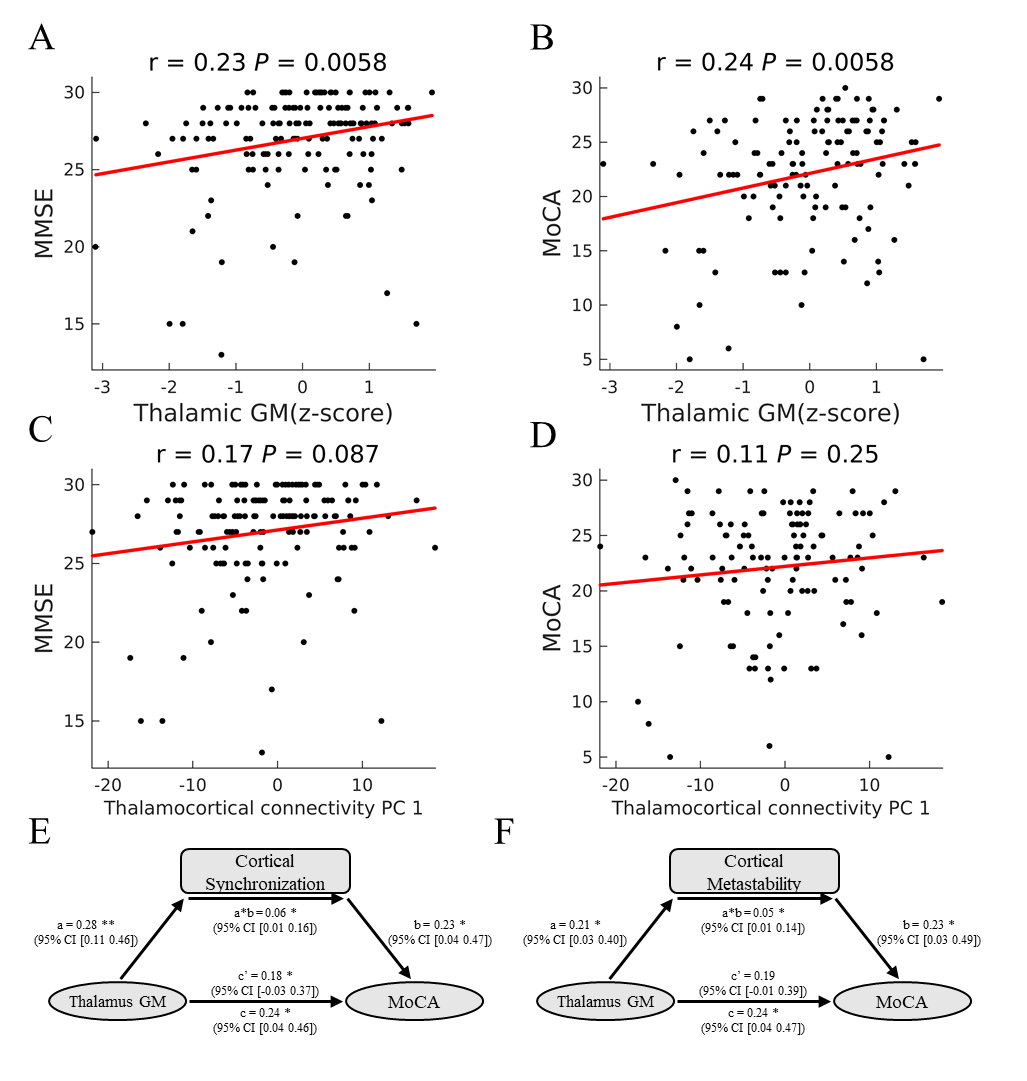


# Figure S7. Relationship between thalamic GM volume and MoCA score at both baseline and follow-up visits using data from the PPMI dataset.


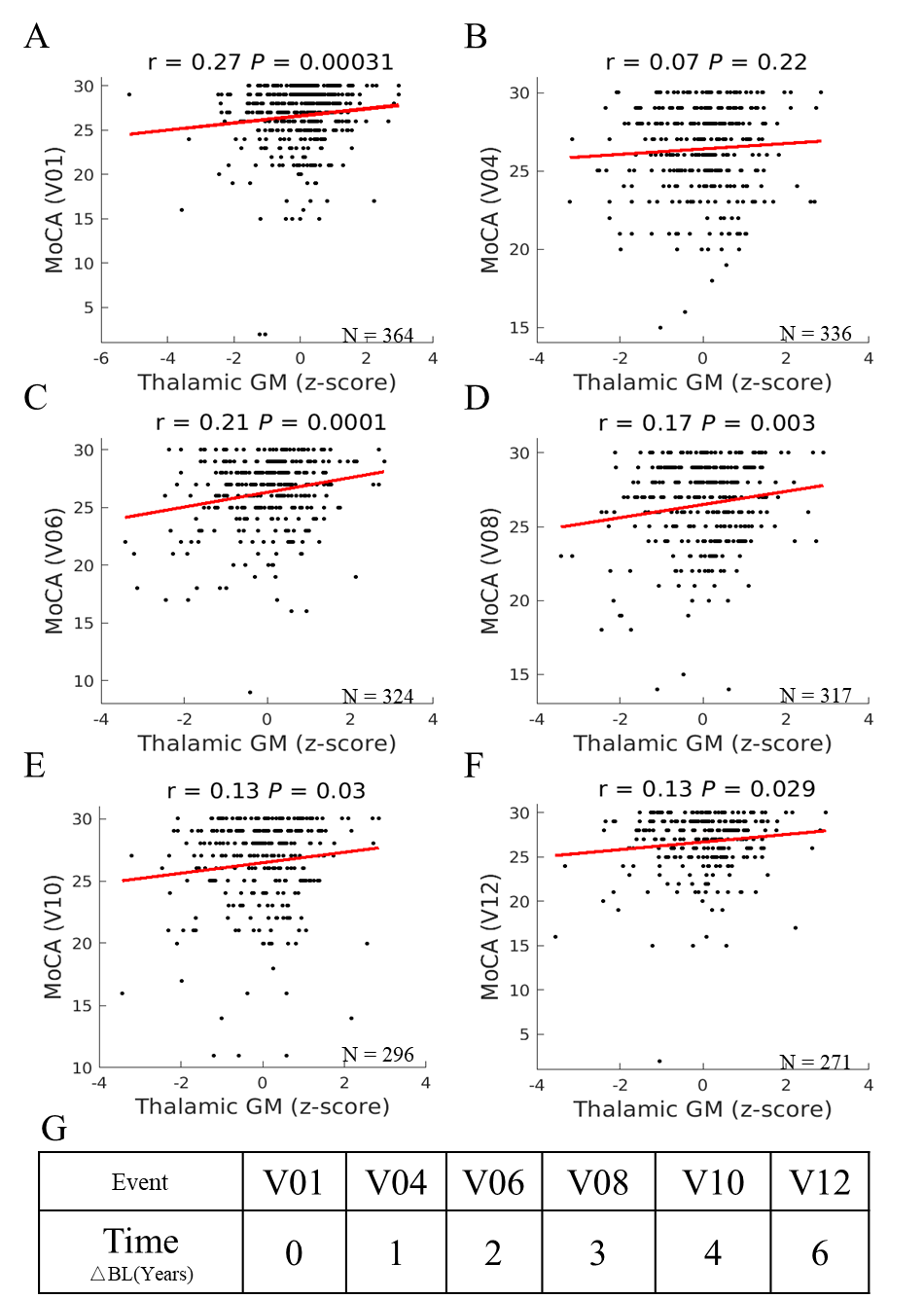


# Figure S8. Relationship between synchronization and metastability within each of the seven RSNs and clinical scores. r values (Pearson’s correlation coefficient) and p values (FDR corrected) are provided. * *p* < 0.05, ** *p* < 0.01, *** *p* < 0.005, FDR corrected.


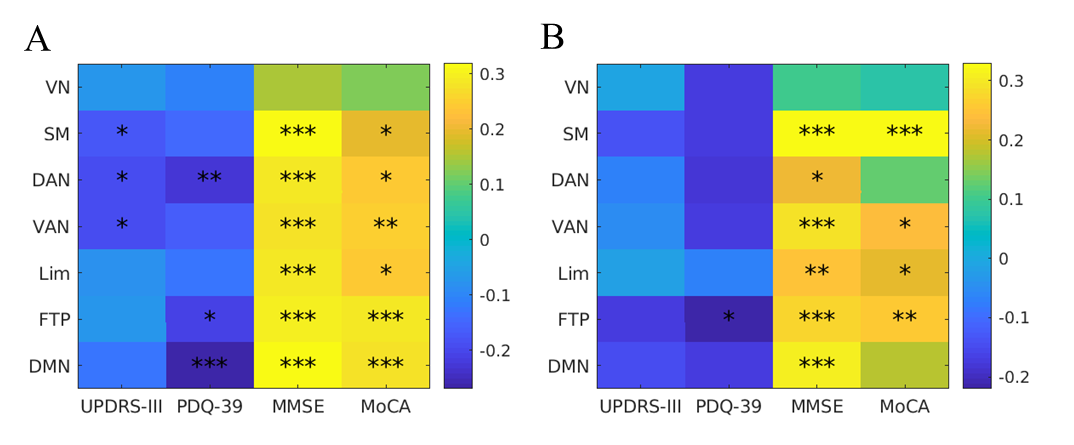


**Figure S9. ﻿﻿Synchronization and metastability within each of the seven RSNs in Parkinson’s disease (PD) ﻿at both the OFF and ON states.** A: The synchronization of DAN, SM, and FTP showed significant increased after levodopa administration. B: The metastability of DMN, FTP, and VN showed significant increased after levodopa administration. DAN: Dorsal attention network; DMN: Default mode network; FTP: Frontoparietal network; Lim: Limbic network; SMN: Somatomotor network; VAN: Ventral attention network; VN: Visual network. NC: Normal controls; PD: Parkinson’s disease. **p* < 0.05, ** *p* < 0.01, *** *p* < 0.005, FDR corrected.


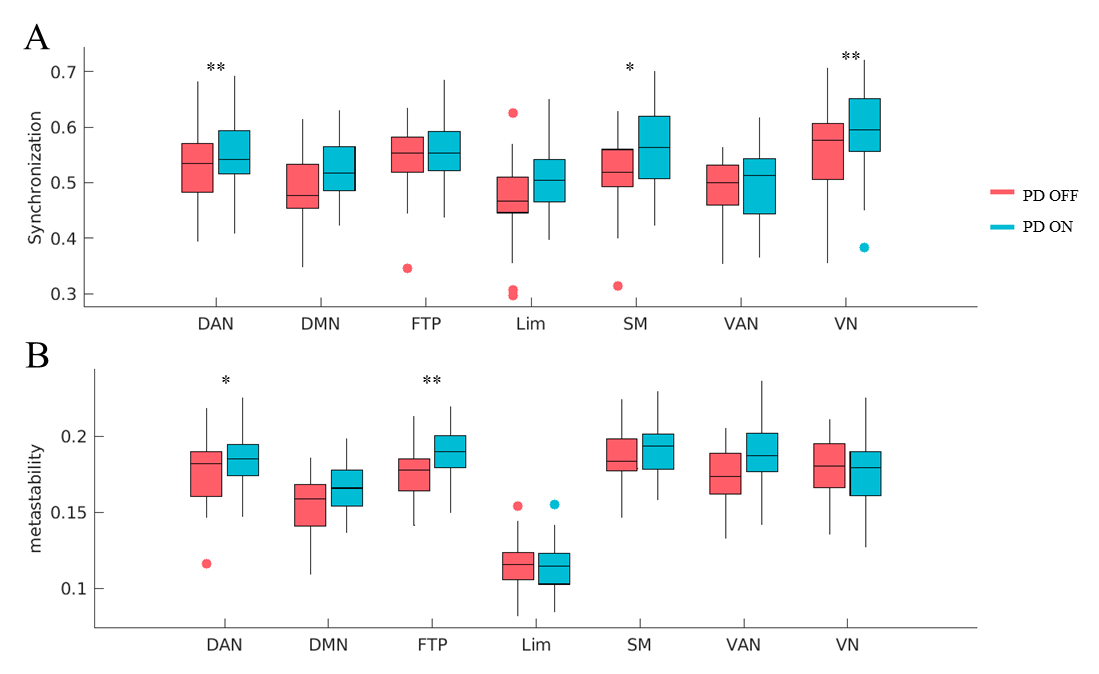


Figure S10. **Subcortical-cortical connectivity in Parkinson’s disease (PD) patients versus normal control (NC) individuals in the seven RSNs.** (A) Putamen-cortical connectivity (average of connectivity between putamen and regions in each network) in PD vs NC in the seven RSNs. (B) Caudate-cortical connectivity (average of connectivity between caudate and regions in each network) in PD vs NC in the seven RSNs. (C) Pallidum-cortical connectivity (average of connectivity between pallidum and regions in each network) in PD vs NC in the seven RSNs. DAN: Dorsal attention network; DMN: Default mode network; FTP: Frontoparietal network; Lim: Limbic network; SMN: Somatomotor network; VAN: Ventral attention network; VN: Visual network. NC: Normal controls; PD: Parkinson’s disease. **p* < 0.05, ** *p* < 0.01, *** *p* < 0.005, FDR corrected.


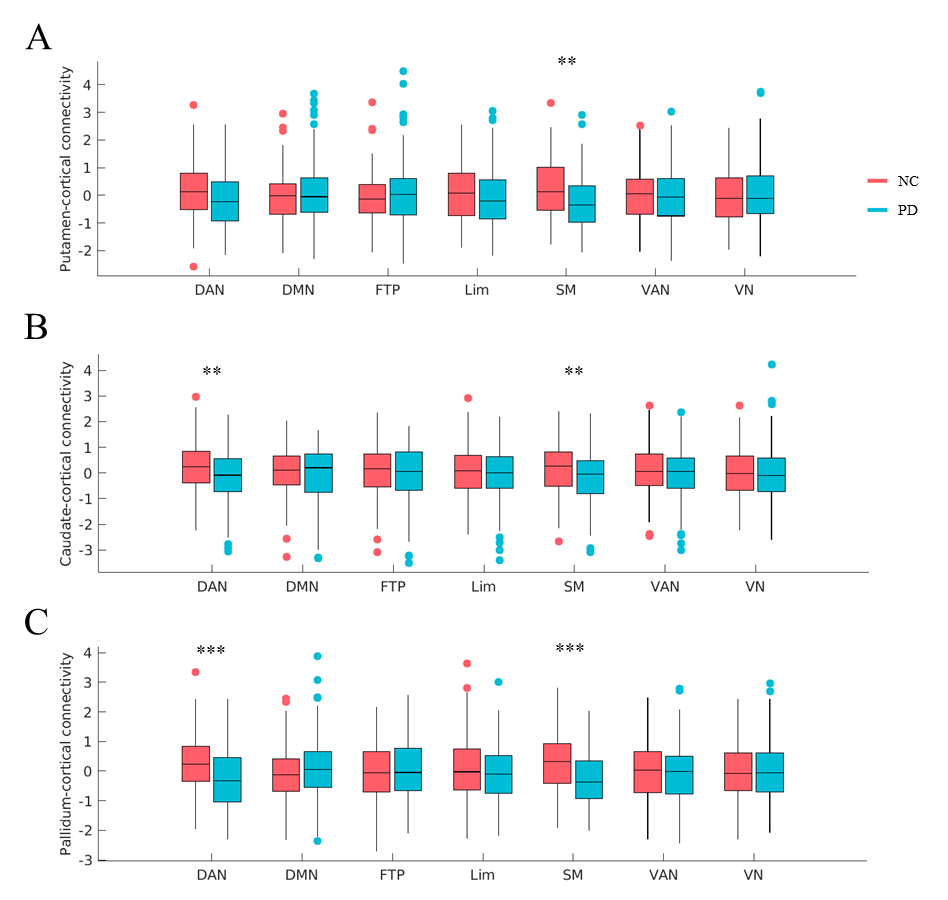

Supplement: Supplementary file 1 — Appendix S1: Supporting Information [file HBM-43-1598-s001.doc]
